# Endoscopic Resection of Zenker's Diverticulum

**DOI:** 10.1155/DTE.7.83

**Published:** 2001

**Authors:** Hideyuki Kataoka, Hiroya Kitano, Masaki Fujimura, Masamitsu Hirano, Takashi Kinoshita, Makoto Hanada, Norikuni Kasuya, Kazutomo Kitajima

**Affiliations:** 1 Department of Otolaryngology Head and Neck Surgery Shiga University of Medical Science Otsu Shiga Japan; 2 Second Department of Surgery Shiga University of Medical Science Otsu Shiga Japan

## Abstract

We report an endoscopically assisted total diverticulectomy for Zenker's diverticulum. Skin incisions were made at the anterior axillary line, the center of the sternum, and the neck as portals for endoscopical instruments. The skin was retracted with hooks which provided an excellent view of the working space. The diverticulum was fully exposed and resected by using a multifire endoscopic stapler. This approach is minimally invasive in comparison with the conventional open cervical approach.


*Diagnostic and Therapeutic Endoscopy*, Vol. 7, pp. 83–87
Reprints available directly from the publisher
Photocopying permitted by license only

© 2001 OPA (Overseas Publishers Association) N.V.
Published by license under
the Harwood Academic Publishers imprint,
part of Gordon and Breach Publishing,
a member of the Taylor & Francis Group.

### *A Case Report*

### Endoscopic Resection of Zenker's Diverticulum

HIDEYUKI KATAOKA[a,*], HIROYA KITANO[a], MASAKI FUJIMURA[b], MASAMITSU HIRANO[b], TAKASHI KINOSHITA[b], MAKOTO HANADA[a], NORIKUNI KASUYA[a] and KAZUTOMO KITAJIMA[a]

[a]*Department of Otolaryngology, Head and Neck Surgery,* [b]*Second Department of Surgery, Shiga University of Medical Science, Otsu, Shiga, Japan*

*(Received 9 February 2001; In final form 9 March 2001)*

**We report an endoscopically assisted total diverticulectomy for Zenker's diverticulum. Skin incisions were made at the anterior axillary line, the center of the sternum, and the neck as portals for endoscopical instruments. The skin was retracted with hooks which provided an excellent view of the working space. The diverticulum was fully exposed and resected by using a multifire endoscopic stapler. This approach is minimally invasive in comparison with the conventional open cervical approach.**

*Keywords*: Diverticulum, Endoscopic surgery, Hooked retractor

### INTRODUCTION

There are a variety of surgical approaches to respect a pharyngo-esophageal diverticulum. Traditionally, an open procedure (diverticulectomy, cricopharyngeal myotomy, or diverticulum suspension) is performed. Dohlman however described an endoscopic approach which avoids an external incision [1]. Using the laryngoscope, the wall between the esophagus and diverticulum is divided with electrocautery. Modifications of this procedure using both lasers [2] and staplers [3] have been developed. However, there are some cases in which it is difficult to use the laryngoscope to expose the diverticulum. Additionally, with the layngoscopic there is always the possibility of dental injury [4]. In this report, we describe a transaxillary endoscopic approach to Zenker's diverticulum as a new, alternative choice for treatment. This approach should result not only in a more rapid postoperative recovery but also in better cosmetic results than open surgical procedures provide.

### CASE REPORT

A 67-year-old woman consulted her family physician complaining a foreign body sensation in her

* Corresponding author. Tel.: +81-77-548-2261. Fax: +81-77-545-7489. E-mail: hkataoka@belle.shiga-med.ac.jp.

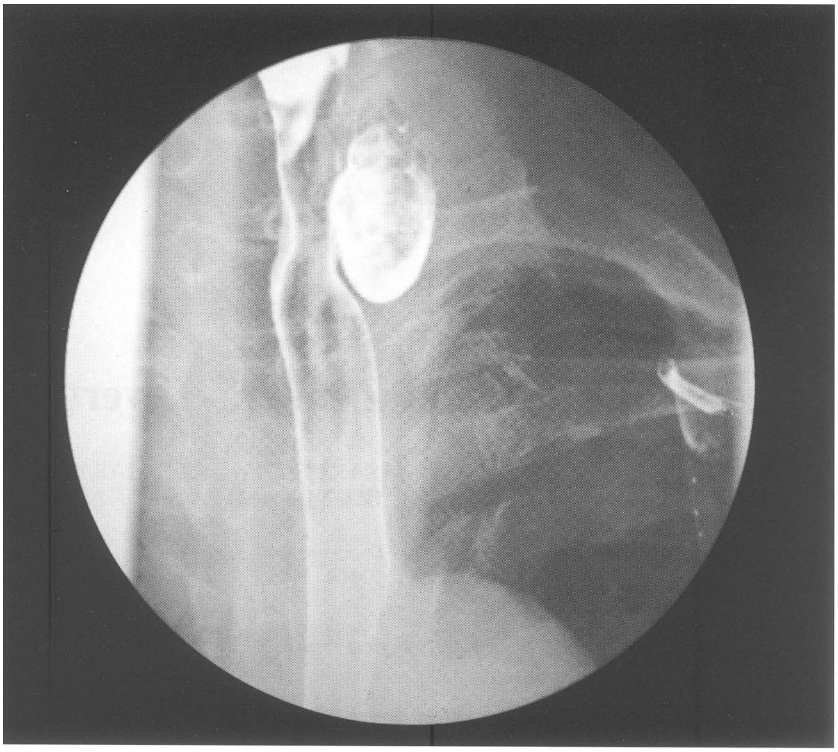

FIGURE 1 Upper gastrointestinal contrast examination showing a large diverticulum.

throat. On an attempt at gastroscopic exam, the scope could not be passed into the esophagus without meeting resistance. Upper gastrointestinal studies showed radiologic evidence of a large esophageal diverticulum (Fig. 1). The patient was referred to our hospital for surgery. An attenuated fiberscope used for viewing the larynx allowed visualization of retained food and debris in the neck of the diverticulum. Informed consent was obtained. The complications of endoscopic neck surgery were discussed according to the standard guidelines of the Ethical Committee of Shiga University of Medical Science Center. The patient opted for endoscopic procedure rather than a traditional open procedure.

### OPERATIVE PROCEDURE

The operation was performed under general anesthesia. Two 10-mm transverse incisions were made bilaterally in the anterior axillary line, for insertion of an endo-retractors, endo-scissors, and endo-peanuts. Additionally, a 10-mm skin incision was placed at the center of the sternum, approximately 10 cm below the upper edge of the clavicle as a portal for the viewing camera; and a 3-mm incision was made in the neck at the level of the thyroid as an opening for the suction/irrigation catheter. At first, blunt dissection was used to make a subcutaneous tunnel under the skin of the chest. Once reaching, the upper edge of the clavicle, flaps were elevated in a subplatysmal plane and raised to the level of the hyoid bone cephalad. The skin was lifted with several hooks (Fig. 2). This retractor provided an excellent view of the working space. The sternohyoid and sternothyroid muscles were retracted medially, and sternocleido mastoid muscle was retracted laterally using vessel tapes passed through the 10-mm axillary skin incisions. After dissecting out the thyroid, the diverticulum was fully exposed posterior to the

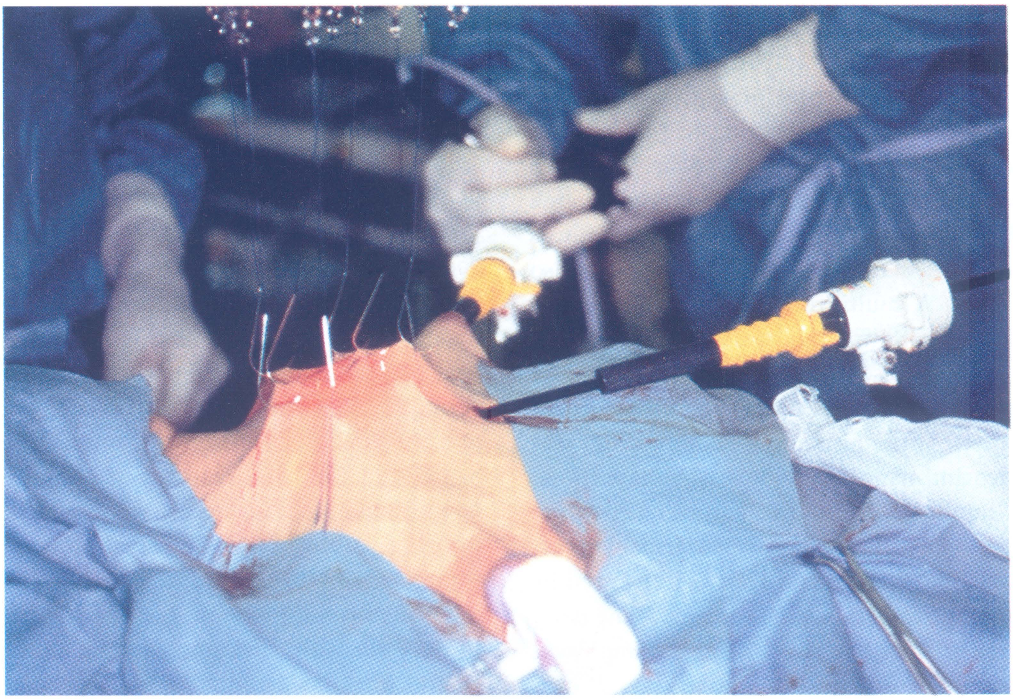

FIGURE 2 The skin was elevated with a hooked retractor.

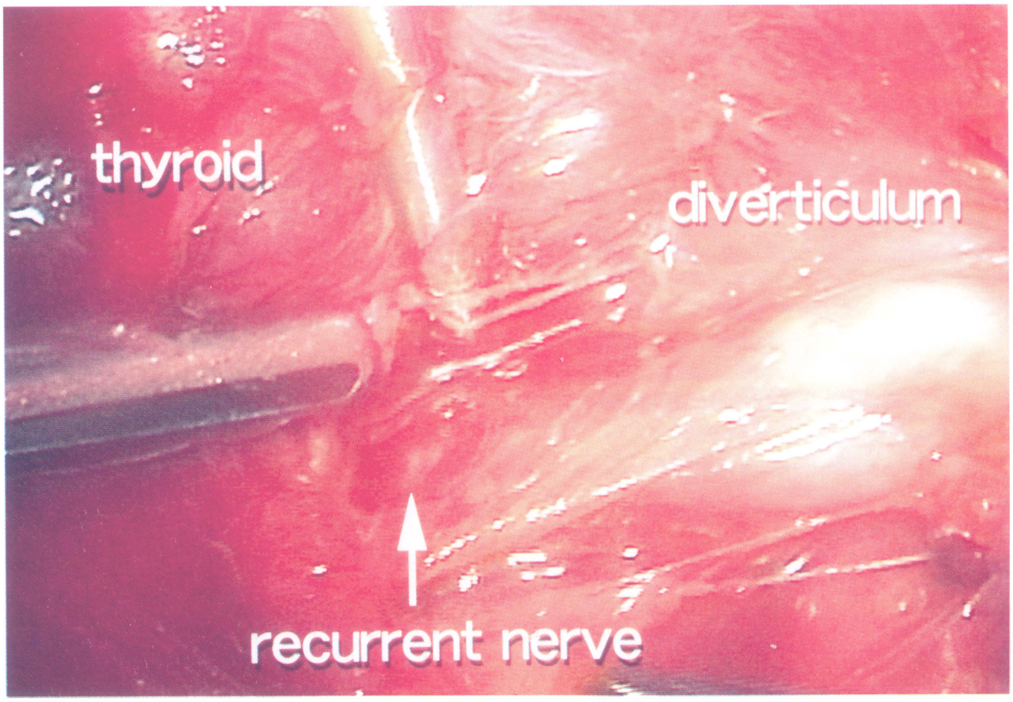

FIGURE 3 The recurrent nerve was identified between the thyroid and the neck of the diverticulum.

left thyroid lobe. The recurrent layngeal nerve was identified, and care was taken to preserve it (Fig. 3). Intraoperative fiberscopic monitoring was used throughout the rase. The location of the diverticulum was identified with the help of the light from the tip of the fiberscope transilluminating through the wall of the esophagus. The fiberscope was helpful not only in finding the diverticulm but also in excising it. A multifire endoscopic stapler, an Endo GIA (AutoSuture, Norwalk, CT, USA), was applied twice to resect the diverticulum (Fig. 4). The specimen was removed via the 10-mm trocar in the center of the sternum. A suction drain was left in place and removed the following day. Blood loss was minimal. The entire surgical procedure took 3 h. Initially, the patient had transient recurrent nerve palsy. We supposed it was influenced by the use of electrical medical instruments. The palsy disappeared 2 months later. Oral feedings was permited on the first postoperative day. It was the first case of endoscopic diverticulectomy in our institution, so careful observation were necessary. The patient was discharged 7 days after surgery. Her symptoms were completely resolved.

### DISCUSSION

The pharyngo-esophageal diverticulum was first described by Ludlow in 1764. Symptoms are variable and may include pyrosis, dysphagia, nocturnal cough, regurgitaion, intermittent aspiration, and weight loss. Patients with minimal symptoms can be treated conservatively. Surgical treatment is recommended for patients with persistent or progressive symptoms [5]. In selecting the appropriate surgical procedure, the size of the diverticulum, its distance from the mouth, and the ability of the patient to withstand surgical complications, should be considered. A laryngoscopic divertic-

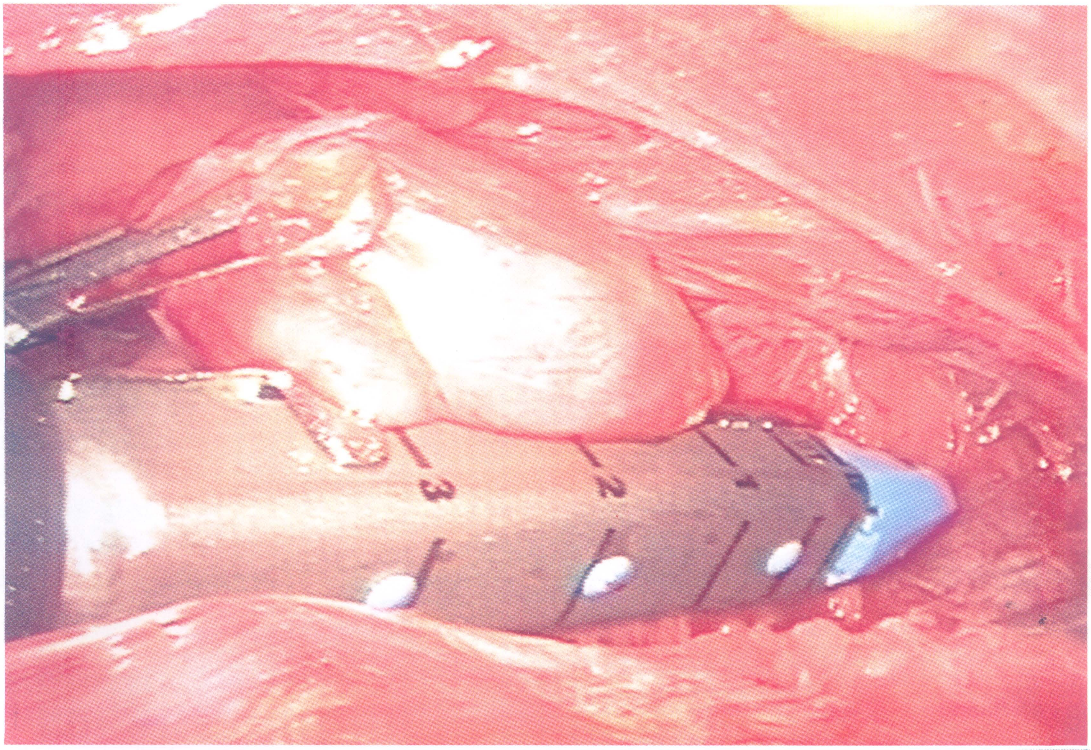

FIGURE 4 The diverticulum was resected with an endoscopic stapler.

ulotomy is minimally invasive, but this procedure is not indicated in all cases. One contraindication for laryngoscopy is the difficulty in exposing the wall between the esophagus and diverticulum. Therefore, the indications of endoscopic neck surgery are limited to cases that are not suitable for laryngoscopic diverticulectomy. In this case, the diverticulum was located deep to the clavicle when viewed on esophagogram, and it was far from the mouth, making it difficult to expose the wall between the esophagus and diverticulum using other methods. The transaxillary endoscopic approach was therefore, the technique of choice. With this approach, a total diverticulectomy could be done without an external cervical approach. The endoscopic incisions were minimal and were subsequently hidden by the patient's undergarments. This approach is less invasive than the conventional cervical approach and may result in less postoperative pain, shorter hospital stays, and earlier resumption of regular activities. In the future, the possibilities for endoscopic neck surgery will be more refined. In conclusion, we recommend our present endoscopic technique as an ideal operative procedure for the treatment of esophageal diverticulum.

### *References*

[1] Dohlman, G. Endoscopic operations for hypopharyngeal diverticula. In *Proceedings of the Fourth International Congress on Otolaryngology: London, 1949*. London: British Medical Association, 1951; **2**: 715–717.

[2] Van Overbeek, J.J. Meditation on the pathogenesis of hypopharyngeal (Zenker's) diverticulum and a report of endoscopic treatment in 545 patients. *Ann. Otol. Rhinol. Laryngol.* 1994; **103**: 178–185.

[3] Scher, R.L. and Richtsmeier, W.J. Endoscopic staple-assisted esophagodiverticulostomy for Zenker's diverticulum. *Laryngoscope* 1996; **106**: 951–956.

[4] Scher, R.L. and Richtsmeier, W.J. Long-term experience with endoscopic staple-assisted esophagodiverticulostomy for Zenker's diverticulum. *Laryngoscope* 1998; **108**: 200–205.

[5] Nguyen, H.C. and Urquhart, A.C. Zenker's diverticulum. *Laryngoscope* 1997; **107**: 1436–1440.

